# Prevalence and clinical characteristics of low skeletal muscle index among adults visiting a health promotion center: Cross-sectional study

**DOI:** 10.1097/MD.0000000000034404

**Published:** 2023-07-21

**Authors:** Jungmi Yun, Ryuk Jun Kwon, Taehwa Kim

**Affiliations:** a College of Nursing, Research Institute of Nursing Science, Pusan National University, Yangsan, Republic of Korea; b Family Medicine Clinic and Research Institute of Convergence of Biomedical Science and Technology, Pusan National University Yangsan Hospital, Yangsan, Republic of Korea; c Division of Pulmonology, Allergy, and Critical Care Medicine, Research Institute for Convergence of Biomedical Science and Technology, Department of Internal Medicine, Pusan National University Yangsan Hospital, Yangsan, Republic of Korea; d Department of Internal Medicine, School of Medicine, Pusan National University, Yangsan-si, Republic of Korea.

**Keywords:** BMI, fat body percent, low skeletal muscle, obesity, sarcopenia

## Abstract

Sarcopenia causes a variety of functional impairments and is associated with all-cause mortality, but once it occurs, it is difficult to treat and reverse. However, the prevalence of sarcopenia in healthy people has never been investigated due to the low awareness of sarcopenia in healthy people. This cross-sectional study was conducted in a single health promotion center from the January 1st 2020 to the December 31st 2021. Adults aged 18 years and older with an Inbody as part of their health checkup were included, and all data was collected from the EMR. Obesity was defined as a body mass index (BMI) of 23 (kg/m^2^) or more by Korean standards, and low skeletal muscle mass was defined as a skeletal muscle index (SMI) of <0.789 for men and <0.512 for women. 60.5% of the total participants (n = 5993) had low skeletal muscle mass. The low SMI group had lower BMI, waist circumference, and abdominal skinfold than the normal SMI group (low SMI group vs normal SMI: BMI; 25.47 ± 2.96 vs 22.98 ± 3.05, *P* < .001, waist circumference; 90.31 ± 8.80 cm vs 82.69 ± 9.71 cm, *P* < .001, abdominal skinfold; 18.78 ± 2.44 mm vs 15.99 ± 2.12 mm, *P* < .001). The body fat percentage was higher in the low SMI group than in the normal SMI group 25.30 ± 6.23% versus 29.82 ± 7.07%, *P* < .001. Triglyceride and uric acid levels were low in the low SMI group (TG; 147.69 ± 97.27 vs 115.86 ± 68.31, *P* < .001, uric acid level; 6.30 ± 1.38 vs 5.23 ± 1.30, *P* < .001) and high-density lipid (HDL) was high (HDL; 53.17 ± 11.41 vs 59.89 ± 14.72, *P* < .001). The odds ratio of low SMI prevalence for age, sex, BMI, fat body percent, and triglycerides relative to normal SMI was 1.05 (*P* = .031), 0.14 (*P* < .001), 0.12 (*P* < .001), 2.05 (*P* < .001), and 0.99 (*P* = .003), respectively. Of those who visited the Health Promotion Center, more than 60% had low SMI identified through Inbody. Low BMI and high body fat percentage increase the risk of low SMI. Compared to normal and low SMI based on obesity, Sex, height, BW, abdominal skinfold, and waist circumflex showed significant P values in both groups. The factors related to low SMI were TG, HDL, and uric acid levels.

## 1. Introduction

Most individuals are unaware of the risk of musculoskeletal diseases, despite being a significant factor in maintaining health. Low muscle mass can lead to sarcopenia, defined as decreased skeletal muscle mass, muscle strength, or physical ability.^[1]^ Some studies have shown that sarcopenia is associated with falls, increased risk of fractures, movement disorders, and decreased activities of daily living, leading to poorer life outcomes.^[2]^ In the end, sarcopenia causes a variety of functional impairments and is associated with all-cause mortality, but once it occurs, it is difficult to treat and reverse.^[3,4]^ Like this, muscle mass is an important survival factor for all diseases, but its diagnostic rate is low. Because healthy people are less aware of sarcopenia caused by low skeletal muscle mass and do not recognize its importance.

Therefore, evaluating for potential musculoskeletal diseases before those become ill is important for health screening. For this reason, many healthcare centers have begun using InBody devices to evaluate muscle mass and body composition.

Although the prevalence of sarcopenia increases with age, several reports have recently reported that the proportion of sarcopenia is considerably higher in older adults and young or middle-aged healthy adults.^[5]^ Several studies have emphasized the importance of early diagnosis of sarcopenia in preventing negative impacts on quality of life.^[6,7]^ In this context, detecting low muscle mass for sarcopenia could be an important health-screening purpose.^[8]^

Based on these trends in the incidence of sarcopenia, we hypothesized that it might be present among people who visited a health promotion center. To our knowledge, the prevalence of sarcopenia sarcopenia caused by low skeletal muscle mass has not been studied in healthy individuals. The purpose of this study was to investigate the prevalence of low skeletal muscle index (SMI) which may be sarcopenia among people who visited a health promotion center, and to identify the characteristics of the low SMI group and factors associated with low SMI. In addition, this study also aimed to investigate the association between obesity and low SMI.

## 2. Methods

### 2.1. Study design

This cross-sectional study was conducted in a single health promotion center from the January 1st 2020 to the December 31st 2021. Body composition was analyzed for adults aged 18 and older who visited a health promotion center and had an Inbody examination. SMI was calculated and divided into a low SMI group and a normal group according to the definition to analyze the difference. A retrospective EMR data collection study was then conducted to identify body composition factors and biomarkers associated with low SMI.

### 2.2. Assessment

Body composition assessments were estimated indirectly using bioelectrical impedance analysis (InBody S10, InBody, Co. Ltd., Seoul, Korea). The parameters included body mass index (BMI), body fat mass, fat-free mass, fat-free mass index, visceral fat area, percent body fat, appendicular skeletal muscle mass, SMI, skeletal muscle mass index, lime mass right arm, lime mass left arm, body trunk, lime mass right leg, and lime mass left leg.^[9,10]^ Basic metabolism ratio was calculated using the following formula: (basic metabolism ratio = 21.6*somatic cell volume [kg] + 370).^[11]^ BMI was calculated using the following formula: weight (kg)/height^2^ (m^2^). BMI was calculated according to the World Health Organization definition of Asian obesity: normal (18.5 ≤ BMI < 23.0 kg/m^2^) or overweight/obese (BMI ≥ 23.0 kg/m^2^).^[12]^

SMI was calculated according to the formula (SMI = skeletal muscles of limbs [kg]*Square of high [m^2^]).^[13]^ According to the criteria for muscle mass loss presented by the foundation for the national institutes of health (FNIH) in 2014,^[14]^ skeletal muscle mass loss was defined as an SMI of <0.789 in men and <0.512 in women.

### 2.3. Clinical data collection

Visitors were classified into 2 groups: those with low SMI and those with normal SMI, per the diagnostic criteria of sarcopenia established by the Asia Working Group (lower than 7.0 and 5.7 kg/m^2^ for men and women, respectively).^[15]^ Visitors were divided into 2 groups according to their initial BMI at admission: obese (≥23 kg/m^2^) and non-obese (<23 kg/m^2^).

Clinical data regarding patient outcomes were retrospectively collected from the medical records. The parameters included sex, age, weight, height, height, waist circumflex, and comorbidities, including hypertension, diabetes mellitus, smoking history, alcohol use, heart rate, systolic blood pressure, and diastolic blood pressure. Laboratory results were investigated according to standard medical guidelines after routine blood collection. Cholesterol, triglycerides, high-density lipid (HDL), low-density lipid (LDL), glucose, insulin, hemoglobin A1c, and uric acid levels were obtained.

### 2.4. Statistical analysis

Data are presented as the mean ± (standard deviation). An independent *t* test was used for continuous variables to evaluate the differences between groups, and the chi-square test was used for categorical variables. Correlations among variables were calculated using Pearson correlation coefficient. Multiple logistic regression analysis was performed to analyze factors related to low SMI using potential factors, including significant variables in univariate analysis. All statistical analyses were performed using the R program version 4.2.2 and SPSS Statistics Software for Windows, Version 27.0. (IBM Corp., Armonk, NY).

### 2.5. Ethical statement

The authors are accountable for all aspects of the work, ensuring that questions related to the accuracy or integrity of any part are appropriately investigated and resolved. The study was conducted in accordance with the principles of the Declaration of Helsinki (revised in 2013).^[16]^ The study procedures were reviewed and approved by the Institutional Review Board of Pusan National University Yangsan Hospital Institutional Review Board.^[1]^ The requirement for informed consent was waived due to the minimal risk associated with a standard-of-care observational study with no interventions.

## 3. Results

### 3.1. Characteristics of patients according to SMI

A total of 6020 patients were enrolled in this study between the 1st of January 2020 and the 31st of December 2021. Among them, 27 visitors without incomplete laboratory, height, and weight data were excluded. The final 5993 patients were included in the analysis. Patients were divided into the low skeletal muscle group of 3627 participants (60.5%) and the normal skeletal muscle group of 2366 participants (39.5%). Table 1 compares the patient characteristics in the normal and low SMI groups.

**Table 1:** Patient characteristics according to SMI (n = 5993).

	Normal SMI (n = 2364, 39.5%)	Low SMI (n = 3626, 60.5%)	*P* value
Age	55.67 ± 11.49	57.46 ± 11.98	<.001
<40	297 (42.8)	397 (57.2)	<.001
40–65	1638 (40.9)	2366 (59.1)	
>65	429 (33.2)	863 (66.8)	
Sex			
Female	161 (6.2)	2450 (93.8)	<.001
Male	2203 (65.2)	1176 (34.8)	
Hight (m)	1.72 ± 0.07	1.62 ± 0.08	<.001
Body weight (kg)	75.06 ± 10.04	60.55 ± 10.72	<.001
BMI	25.47 ± 2.96	22.98 ± 3.05	<.001
Waist circumflex (cm)	90.31 ± 8.80	82.69 ± 9.71	<.001
Percent body fat (%)	25.30 ± 6.23	29.82 ± 7.07	<.001
Abdominal skinfold	18.78 ± 2.44	15.99 ± 2.12	<.001
Hypertention	635 (45.4)	764 (54.6)	<.001
DM	264 (41.6)	371 (58.4)	.252
Heart rate	75.47 ± 12.62	77.76 ± 12.93	<.001
sBP	123.41 ± 12.55	120.26 ± 14.34	<.001
dBP	81.64 ± 8.99	78.04 ± 9.58	<.001
Smoking			
Never	1565 (33.2)	3149 (66.8)	<.001
Current	704 (65.2)	375 (34.8)	
Unknown	95 (48.2)	102 (51.8)	
Alcohol			
No	1186 (31.5)	2577 (68.5)	<.001
Yes	1178 (52.9)	1047 (47.1)	

BMI *=* body mass index, dBP = diastolic blood pressure, DM = diabetes mellitus, sBP = systolic blood pressure, SMI = skeletal muscle mass.

There were significant differences in age (mean age) and sex between the groups (normal SMI vs low SMI; age: 55.67 ± 11.48 years vs 57.46 ± 11.98 years, *P* < .001, sex: male 93.2% vs 32.5%). There was also a difference in the incidence rate between the 2 groups by age when comparing the groups <40, 40 to 65, and 65 years or older. The BMI was 25.46 ± 2.96 kg/m^2^ in the normal SMI group, while the low SMI group was 22.98 ± 3.05 kg/m^2^, indicating a significant *P* value (*P* < .001). The waist circumference was 90.31 ± 8.80 cm in the normal SMI group, while the low SMI group was 82.69 ± 9.71 cm, indicating a significant *P* value (*P* < .001). The percent body fat was 25.30% ± 6.23% in the normal SMI group, while the low SMI group was 29.82% ± 7.09%, indicating a significant *P* value (*P* < .001). Visceral fat thickness was 18.78 ± 2.44 cm in the normal SMI group, while the low SMI group was 15.99 ± 2.12 cm, indicating a significant *P* value (*P* < .001). Hypertension of comorbidity was higher normal SMI than low SMI (normal SMI vs low SMI; 26.8% vs 21.1%, *P* < .001), DM showed no significant difference. Heart rate, sBP, and dBP were significantly different between the 2 groups (normal SMI vs low SMI; heart rate: 75.47 ± 12.615 beat/min vs 77.75 ± 12.933 beat/min, *P* < .001; sBP:120.340 ± 12.551 mm Hg vs 120.26 ± 14.342 mm Hg, *P* < .001; dBP:81.65 ± 8.991 mm Hg vs 78.04 ± 9.578 mm Hg, *P* < .001). Smoking and alcohol consumption differed significantly between the 2 groups (normal vs low SMI: Smoking, *P* < .001; alcohol use, *P* < .001).

### 3.2. Difference between the 2 groups in laboratory findings

Table 2 compares the patients’ blood and biochemical parameters between the normal and low SMI groups. The cholesterol was 199.90 ± 43.72 mg/dL in the normal SMI group, the low SMI group showed 201.24 ± 49.08 mg/dL, and there was no significant *P* value between the 2 groups (*P* = .267). The triglyceride levels in the 2 groups were 147.67 ± 97.24 mg/dL in the normal SMI group and 115.96 ± 68.58 mg/dL in the low SMI group, indicating significant differences between the 2 groups (*P* < .001). HDL cholesterol and glucose in the normal SMI group were 122.32 ± 31.19 mg/dL and 109.70 ± 26.65 mg/dL, respectively. HDL cholesterol and glucose in the low SMI group were 121.39 ± 35.41 mg/dL and 107.30 ± 27.21 mg/dL, respectively (normal SMI vs low SMI: HDL, *P* < .001; glucose, *P* < .001). However, LDL cholesterol was not statistically significant between the 2 groups (normal SMI vs low SMI: LDL; 122.32 ± 31.19 mg/dL vs 121.39 ± 35.41 mg/dL, *P* = .282). Insulin and HbA1c showed no difference between the 2 groups (normal SMI vs low SMI: insulin, 6.14 ± 4.43 mlU/L vs 5.80 ± 9.91 mlU/L, *P* = .133; HbA1c, 5.75% ± 0.79% vs 5.73% ± 0.73%, *P* = .233). Uric acid for the normal and low SMI groups was 6.30 ± 1.38 mg/dL vs 5.23 ± 1.30 mg/dL, respectively; the 2 groups had a significant *P* value.

**Table 2:** Difference in laboratory findings between the 2 groups (n = 5993).

	Total		
	Normal SMI (n = 2366, 39.5%)	Low SMI (n = 3627, 60.5%)	*P* value
Cholesterol	199.89 ± 43.73	201.23 ± 49.08	.280
Triglyceride	147.69 ± 97.27	115.86 ± 68.31	<.001
HDL	53.17 ± 11.41	59.89 ± 14.72	<.001
LDL	122.32 ± 31.19	121.38 ± 35.40	.288
Glucose	109.72 ± 26.65	107.30 ± 27.21	.001
Insulin	6.14 ± 4.43	5.80 ± 9.91	.132
HbA1c	5.75 ± 0.79	5.73 ± 0.73	.218
Uric acid	6.30 ± 1.38	5.23 ± 1.30	<.001

HDL = high-density lipid, LDL = low-density lipid, SMI = skeletal muscle index.

### 3.3. Subgroup analysis according to obesity

Table 3 shows the subgroup analysis according to obesity status. Low SMI was higher in the non-obese group than in the obesity group (81.3% vs 46.9%). In the non-obese group, there was no significant difference in age according to skeletal muscle mass. In the obesity group, age was significantly different only in the obesity group. Regarding sex differences, both women and men in the non-obese group accounted for a higher rate of low SMI; However, in the obese group, only women had a higher proportion of low SMI. Hypertension did not show a significant difference in the underlying disease in either group. However, DM was associated with a high proportion of low SMI in both the non-obesity and obesity groups.

**Table 3:** Analysis according to obesity levels (n = 5993).

	Non-obesity (n = 2370)			Obesity (n = 3620)		
	Normal SMI (n = 442, 18.6%)	Low SMI (n = 1928, 81.4%)	*P* value	Normal SMI (n = 1922, 53.1%)	Low SMI (n = 1698, 46.9%)	*P* value
Age	56.10 ± 11.83	56.07 ± 12.02	.965	55.58 ± 11.41	59.04 ± 11.74	<.001
<40	54 (17.8)	249 (82.2)	.924	243 (62.1)	148 (37.9)	<.001
40–65	298 (18.8)	1290 (81.2)		1340 (55.5)	1076 (44.5)	
>65	90 (18.8)	389 (81.2)		339 (41.7)	474 (58.3)	
Sex						
Female	11 (0.8)	1445 (99.2)	<.001	150 (13.0)	1005 (87.0)	<.001
Male	431 (47.2)	483 (52.8)		1772 (71.9)	693 (28.1)	
Hight (m)	1.73 ± 0.06	1.62 ± 0.07	<.001	1.71 ± 0.07	1.62 ± 0.09	<.001
Body weight (kg)	65.63 ± 5.36	54.33 ± 6.31	<.001	77.23 ± 9.60	67.62 ± 10.31	<.001
BMI	21.82 ± 0.95	20.74 ± 1.53	<.001	26.30 ± 2.61	25.52 ± 2.25	<.001
Waist circumfllex (cm)	81.22 ± 4.96	76.66 ± 6.33	<.001	92.40 ± 8.14	89.55 ± 8.22	<.001
Percent body fat (%)	18.93 ± 3.77	27.00 ± 5.90	<.001	26.77 ± 5.74	33.04 ± 6.93	<.001
Abdominal skinfold	16.49 ± 1.45	15.10 ± 1.70	<.001	19.31 ± 2.31	17.59 ± 1.82	<.001
Hypertention	76 (22.0)	270 (78.0)	.087	559 (53.1)	494 (46.9)	.995
DM	47 (25.5)	137 (74.5)	.012	217 (48.1)	234 (51.9)	.023
Heart rate	75.64 ± 12.96	78.26 ± 13.17	<.001	75.43 ± 12.54	77.19 ± 12.64	<.001
sBP	119.16 ± 12.88	117.03 ± 13.56	.003	124.38 ± 12.27	123.94 ± 14.33	.313
dBP	79.15 ± 9.14	76.33 ± 9.59	<.001	82.22 ± 8.86	80.00 ± 9.19	<.001
Smoking						
Never	283 (14.2)	1705 (85.8)	<.001	1282 (47.0)	1444 (53.0)	<.001
Current	142 (42.5)	192 (57.5)		562 (75.4)	183 (24.6)	
Unknown	17 (35.4)	31 (64.6)		78 (52.3)	71 (47.7)	
Alcohol						
No	225 (14.1)	1370 (85.9)	<.001	961 (44.3)	1207 (55.7)	<.001
Yes	217 (28.0)	557 (72.0)		961 (66.2)	490 (33.8)	

BMI *=* body mass index, dBP = diastolic blood pressure, DM = diabetes mellitus, FFMI = fat free mass index, LMLA = lean mass Left arm, LMLL = lean mass left leg, LMRA = lean mass right arm, LMRL = lean mass right leg, sBP = systolic blood pressure, SMI = skeletal muscle mass, SSMI = skeletal muscle mass index.

Smoking and drinking differed regardless of obesity status. Among the body composition variables, all variables except lime mass right leg showed significant differences between the normal and low SMI groups, regardless of obesity. There were differences between the 2 groups in cholesterol, triglyceride, HDL, glucose, and uric acid levels according to low SMI in the non-obese group. In the obese group, cholesterol, triglyceride, HDL, HbA1c, and uric acid levels differed between the 2 groups according to low SMI (Table 4).

**Table 4:** Laboratory findings according to obesity levels (n = 5993).

	Non-obesity (n = 2370)			Obesity (n = 3620)		
	Normal SMI (n = 442, 18.6%)	Low SMI (n = 1928, 81.4%)	*P* value	Normal SMI (n = 1922, 53.1%)	Low SMI (n = 1698, 46.9%)	*P* value
Cholesterol	197.57 ± 41.41	204.81 ± 45.63	.002	200.42 ± 44.24	197.16 ± 52.44	.043
Triglycerin	114.82 ± 81.51	100.96 ± 52.81	<.001	155.25 ± 99.04	132.80 ± 79.15	<.001
HDL	56.53 ± 11.47	64.27 ± 14.54	<.001	52.40 ± 11.25	54.91 ± 13.28	<.001
LDL	119.83 ± 30.27	121.09 ± 33.71	.470	122.90 ± 31.38	121.70 ± 37.23	.294
Glucose	107.07 ± 30.62	103.32 ± 25.24	.007	110.32 ± 25.62	111.81 ± 28.64	.099
Insulin	4.24 ± 2.30	4.67 ± 5.89	.166	6.56 ± 4.67	7.05 ± 12.84	.135
HbA1c	5.62 ± 0.73	5.61 ± 0.66	.748	5.78 ± 0.80	5.86 ± 0.78	.003
Uric acid	6.04 ± 1.26	4.96 ± 1.19	<.001	6.36 ± 1.40	5.54 ± 1.35	<.001

HDL = high-density lipid, LDL = low-density lipid, SMI = skeletal muscle index.

### 3.4. Prevalence of low SMI according to BMI and body fat quartiles

**Figure 1.**
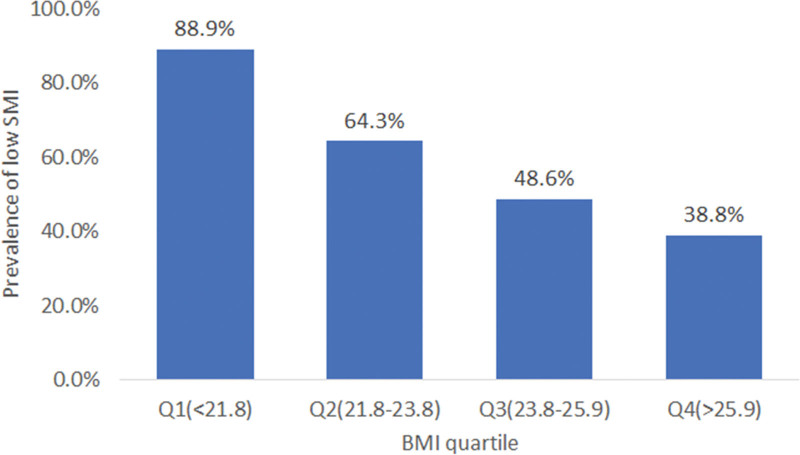
Prevalence of low skeletal muscle index (SMI) according to body mass index (BMI) quartile and body fat quartile.

The prevalence of low SMI according to BMI and body fat percentage quartile is shown in Figure 1. Low SMI prevalence according to BMI was highest in Q1 and gradually decreased in Q2, Q3, and Q4. This means that the lower the BMI, the more low SMI occurs (low SMI prevalence: Q1, 88.9%; Q2, 64.2%, Q3, 48.7%; Q4, 38.8%). The prevalence of low SMI according to the body fat quartile, was highest in Q1 and lowest in Q3, Q2, and Q4, in that order. Although no gradual trend was observed according to the body fat quartile, the results showed that Q1 was the highest and Q4 was the lowest (low SMI prevalence: Q1, 65.9%; Q2, 59.4%; Q3, 61.1%; Q4, 55.2%).

### 3.5. Logistic regression for low SMI influencing factors

Finally, we performed multiple logistic regression analyses for low SMI (Table 5). The odds ratio of low SMI prevalence for age, sex, BMI, fat body percent, and triglyceride levels relative to normal SMI was 1.05 (*P* = .031), 0.14 (*P* < .001), 0.12 (*P* < .001), 2.05 (*P* < .001), and 0.99 (*P* = .003), respectively. In the non-obese group, the odds ratios of low SMI prevalence for sex, BMI, body fat percentage, and HDL compared to normal SMI were 0.18 (*P* = .016), 0.09 (*P* < .001), 2.04 (*P* < .001), 1.04 (*P* = .008), respectively. In the obesity group, the odds ratios for age, sex, BMI, fat body percentage, and triglycerides were 1.04 (*P* = .016), 0.27 (*P* = .002), 0.12 (*P* < .001), 2.37 (*P* < .001), and 0.99 (*P* = .007), respectively.

**Table 5:** Logistic regression for low skeletal muscle index (SMI) influencing factors.

	Total		Non-obesity		Obesity	
	OR (95% CI)	*P* value	OR (95% CI)	*P* value	OR (95% CI)	*P* value
Age (yr)	1.02 (1.00, 1.05)	.031			1.04 (1.01, 1.07)	.016
Sex (Ref: Female)						
Male	0.14 (0.07, 0.29)	<.001	0.18 (0.04, 0.72)	.016	0.27 (0.12, 0.63)	.002
BMI	0.12 (0.09, 0.16)	<.001	0.09 (0.05, 0.16)	<.001	0.12 (0.08, 0.19)	<.001
Fat body percentage	2.05 (1.84, 2.29)	<.001	2.04 (1.73, 2.41)	<.001	2.37 (1.98, 2.83)	<.001
Triglycerin	0.99 (0.99, 1.00)	.003			0.99 (0.99, 1.00)	.007
HDL			1.04 (1.01, 1.08)	.008		

BMI = body mass index, HDL = high-density lipid.

## 4. Discussion

In this study, we investigated adults who visited the Health Promotion Center. We identified the prevalence and clinical characteristics of the low SMI group and investigated the factors that may affect low SMI. 60.5% of all participants who had health checkups were screened into the low SMI group. The proportion of the low SMI group was higher than normal SMI among those aged 65 or older, and 67.5% of women had low SMI. These participants had not been diagnosed with sarcopenia. These findings suggest that many adults might have a low SMI, but remain unaware of it. This implies a low SMI may be underdiagnosed or not identified early enough.

The body composition analysis results showed that the low SMI group had a lower BMI than the normal SMI group. Additionally, the low SMI group had a lower waist circumference and abdominal skinfold. The results were the same, regardless of the presence of obesity. However, body fat percentage was higher in the low SMI group than in the normal SMI group. This showed that the low SMI group had high body fat, even in the non-obese group. The prevalence of sarcopenia decreased as BMI increased. This finding suggests that groups with high body fat percentages and low BMI are at an increased risk of developing low SMI. Therefore, low SMI in patients should not be evaluated solely on BMI; it should be considered in combination with body fat percentage.

Triglyceride and uric acid levels were low, and HDL levels were high in the low SMI group. The same result was obtained when the obesity rate was used. This is notable compared with previous studies on the relationship between sarcopenia and metabolic syndrome. Previous studies have shown that the prevalence of metabolic syndrome factors such as abdominal obesity, hypertension, hyperglycemia,^[17–19]^ and low HDL cholesterolemia is significantly increased in the low SMI prevalence group. Low SMI is an independent risk factor for metabolic syndrome, and low muscle mass reportedly increases the probability of metabolic syndrome as the grade progresses. However, our results confirmed higher HDL levels in the low SMI group; triglycerides and uric acid were identified as new related factors. It can be assumed that the results may differ because the analysis was performed for the health checkup group and not the patient target. However, further studies on related factor analysis and risk analysis through PSM will be needed to determine exactly why, unlike the previous results, low triglyceride and high HDL levels occur in the low SMI group. What is certain is that a low SMI is an associated factor in metabolic syndrome, which is an important consideration for health management. Therefore, low SMI is not only useful for the initial diagnosis of sarcopenia, but it can also cause various diseases, such as metabolic diseases; therefore, its diagnostic value is important.

In comparison by sex, 93.8% of women were found to have a low SMI. We applied a low SMI criterion to the existing FNIH data. The Asian Working Group for Sarcopenia,^[15]^ the European Working Group on Sarcopenia,^[20]^ FNIH,^[14]^ and the International Sarcopenia Working Group (IWGS)^[21]^ report different cutoff values for sarcopenia. Therefore, to obtain an accurate value according to the target, verification by race, age, and sex in each country is being attempted in various ways. When our results were also applied to FNIH, more than 90% of women were diagnosed with low SMI. This is evidence that domestic standards for low SMI by age and sex are needed, and further verification is necessary.

The present study has several limitations. This study was conducted at a single center and primarily reflected the characteristics of the target group. In addition, since this was a cross-sectional study, it had limitations in explaining temporal causality. Therefore, prospective studies on exercise habits, diet, and nutritional status are required. Skeletal muscle mass was measured using bioelectrical impedance analysis alone, despite the dominance of DEXA for estimating muscle mass. However, the validity and reproducibility of the device used in our study were verified and correlated well with DEXA results.

This study highlights the need to increase awareness and detect low skeletal muscle mass (SMI) in adults, as many people may have low SMI without being diagnosed or recognized. Healthcare professionals should consider assessing body composition beyond BMI alone by incorporating body fat percentage, waist circumference, and abdominal skinfolds accurately evaluate muscle mass and identify individuals at risk for low SMI. Public health initiatives should focus on maintaining and improving skeletal muscle mass, especially in high-risk groups such as older adults and women, by promoting regular exercise, proper nutrition, and an overall healthy lifestyle. The findings emphasize the importance of considering low SMI as a potential risk factor for metabolic syndrome and other related diseases. It emphasizes the need for comprehensive healthcare strategies that target both muscle and metabolic health and the development of public health initiatives that can effectively identify and manage low SMI, potentially improving health outcomes in the population.

## 5. Conclusion

In conclusion, this study found an association between low SMI and obesity in the healthy group, with low SMI being higher in the non-obese group than in the obese group. This suggests that a lower SMI is associated with a higher risk of sarcopenia, even in a healthy population. The body composition analysis results emphasize the importance of assessing the association between BMI and body fat percentage rather than BMI alone in a healthy group. Low BMI and high body fat percentage increase the risk of developing sarcopenia. In addition, the prevalence of metabolic syndrome components is high in the non-obese and low SMI groups; they may be vulnerable to metabolic syndrome and chronic diseases. Therefore, early and appropriate management is needed.

## Acknowledgments

This study was supported by Research institute for Convergence of Biomedical Science and Technology, Pusan National University Yangsan Hospital (20-2022-001).

## Author contributions

**Conceptualization:** Jungmi Yun, Ryuk Jun Kwon, Taehwa Kim.

**Data curation:** Ryuk Jun Kwon, Taehwa Kim.

**Validation:** Jungmi Yun, Ryuk Jun Kwon.

**Writing – original draft:** Jungmi Yun.

**Writing – review & editing:** Taehwa Kim.
